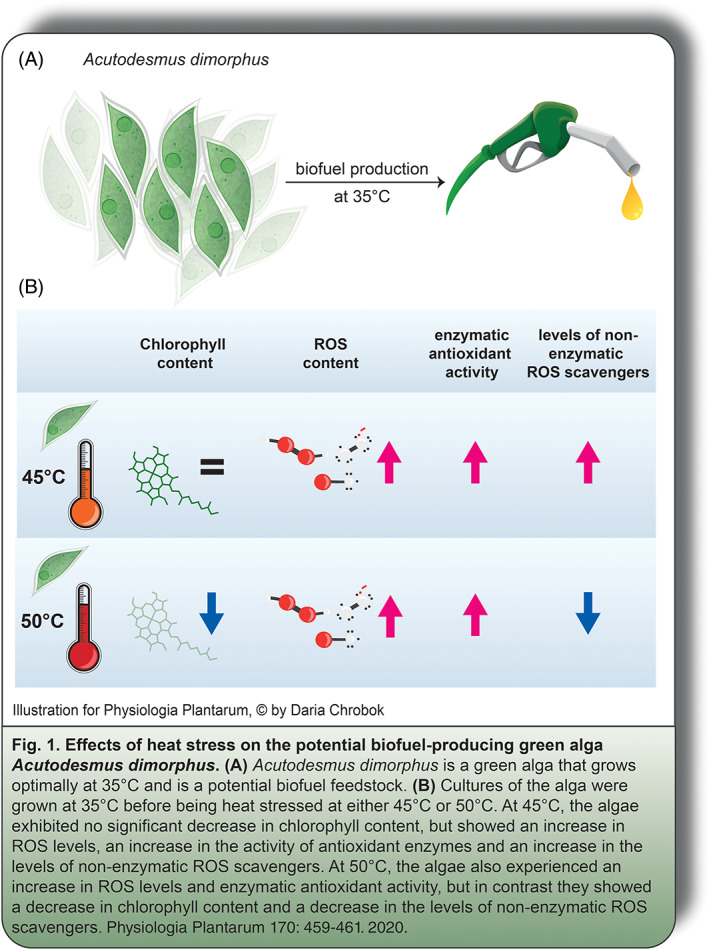# The temperatures, they are a‐changin': How to produce biofuels in a warming world

**DOI:** 10.1111/ppl.13257

**Published:** 2020-11-21

**Authors:** Robert H. Calderon

**Affiliations:** ^1^ Department of Plant Physiology Umeå University Umeå Sweden

**One promising way for humans to sustainably produce energy is to ask photosynthetic organisms to do it for us. Many researchers are searching for naturally occurring algal strains that produce high levels of compounds that can be directly used as biofuels. Whether these strains are used to produce hydrogen gas or liquid products like ethanol, it is important that the cultivation of these strains can be optimized to maximize fuel production. One limitation on the commercial growth of algae is that they are often cultivated outdoors, making them subject to changing weather and temperature conditions (Béchet et al.** **2017** **). In order to understand how a given strain might respond to such temperature changes out in the real world, it is important to first see how they respond in the laboratory.**

In this issue of *Physiologia Plantarum*, Choksi et al. (2020) have sought to measure the ability of the alga *Acutodesmus dimorphus* to adapt to short periods of high temperature stress. This alga was previously found to produce optimal levels of potential biofuels at 35°C (Figure 1a), making it ideal for cultivation in parts of the world with warmer climates (Chokshi et al. 2015). To test the ability of *A. dimorphus* to respond and adapt to temperature stress, the authors first grew the strain at 35°C and then raised the temperature to either 45 or 50°C. The algal response was then measured using markers of photosynthesis as well as the indicators of the production and detoxification of reactive oxygen species (ROS).

The authors found that after 8 h, the levels of the photosynthetic pigment chlorophyll began to significantly decrease in the strain grown at 50°C, while chlorophyll levels remained relatively unchanged at 45°C (Figure 1b). A rapid reduction in chlorophyll content is a strong signal that the cells are undergoing high levels of heat stress (Berry and Bjorkman 1980). In practical terms, the strain seems capable of withstanding exposure to 45°C, but prolonged exposure to temperatures of 50°C are likely to substantially reduce, if not eliminate, potential biofuel production.

Heat stress in algae often leads to increased production of dangerous ROS molecules like hydrogen peroxide and singlet oxygen. Measurements of ROS levels at 45 or 50°C revealed that both temperatures led to a burst of these ROS molecules inside the cells (Figure 1b). The samples that were moved to 45°C appeared capable of eventually detoxifying some of these ROS molecules while those moved to 50°C did not. Cells can mitigate the damaging effects of ROS through the activity of ROS‐detoxifying enzymes (like catalase, superoxide dismutase, or ascorbate peroxidase) or by producing non‐enzymatic ROS scavengers like polyphenols or proline. When the authors measured the activity of ROS‐detoxifying enzymes, they found an increase in their activity at both temperatures. However, when they measured levels of non‐enzymatic ROS scavengers (polyphenols and proline), they observed that longer growth times at 50°C led to lower amounts of these ROS scavenging molecules, indicating a reduced capacity to eliminate ROS.

The implications of this study for potential biofuel production are important to consider. The most obvious of these is that growth of *Acutodesmus dimporphus* for the generation of bioenergy should be done in places that have an average temperature of 35°C but that do not experience temperatures of 50°C or higher. However, temperature changes can be rapid and unpredictable, especially as the global climate continues to change, so it would also be wise for potential algal farmers to consider alternative strategies to cope with potential heat stress.

Estimates for the cost of constructing massive refrigerated outdoor pools of biofuel‐producing algae are hard to come by, but it is probably fair to assume that these would be enormously expensive. A different strategy for dealing with heat stress would be to bioengineer heat‐resistant strains of algae like *A. dimorphus*. According the results presented by the Chokshi *et al*., a good place to begin would be to attempt to increase levels of non‐enzymatic antioxidants like proline and polyphenols since these compounds decrease after 24 h of heat stress. Perhaps the key to unlocking the potential of algal strains for the production of biofuels will be to employ a combination of the above strategies.

Clearly much work is left to be done before humans are able to sustainably generate bioenergy, but the insights into the physiology of biofuel producing microorganisms presented by Chokshi *et al*. take us a step closer toward achieving that goal.